# US FDA Approval of Pediatric Artificial Intelligence and Machine Learning–Enabled Medical Devices

**DOI:** 10.1001/jamapediatrics.2024.5437

**Published:** 2024-12-16

**Authors:** Ryan C. L. Brewster, Matthew Nagy, Susmitha Wunnava, Florence T. Bourgeois

**Affiliations:** 1 Department of Pediatrics, Boston Children’s Hospital, Harvard Medical School, Boston, Massachusetts; 2 Harvard-MIT Center for Regulatory Science, Harvard Medical School, Boston, Massachusetts; 3 Pediatric Therapeutics and Regulatory Science Initiative, Computation Health Informatics Program, Boston Children's Hospital, Boston, Massachusetts

## Abstract

This cross-sectional study analyzes the availability of artificial intelligence and machine learning–enabled devices authorized for children by the US Food and Drug Administration (FDA) and assesses reporting of algorithm validation in the pediatric population.

Medical technologies for children have historically lagged behind those for adults.^1^ Artificial intelligence–enabled and machine learning–enabled (AI/ML) medical devices represent a rapidly growing product type with unique requirements for extension to pediatric patients. Specifically, device algorithms require validation with datasets that include children to ensure effective application in the intended population.^2^ However, while the US Food and Drug Administration (FDA) stipulates that AI/ML algorithms undergo clinical validation, there are no requirements for manufacturers to specify whether testing included pediatric individuals or for device labels to present standard information on the age of patients for which a device was developed.^3,4^ Accordingly, this cross-sectional study aimed to quantify the availability of AI/ML-enabled devices authorized for children and to assess reporting of algorithm validation in the pediatric population.

## Methods

All AI/ML-enabled medical devices marketed in the US from November 1995 through March 2024 were identified from the FDA’s website.^5^ Based on FDA approval documents, each device was classified as labeled for use in children (<18 years) if approval was explicitly mentioned for this age group or as authorized for adults only if children were excluded or the label was silent on pediatric use. Of note, we selected an age range (<18 years) that aligns with the conventional clinical definition for pediatrics, although the FDA classifies pediatric patients as younger than 22 years in the regulation of medical devices. Devices with missing approval documents (n = 6) were excluded. The description of the clinical performance testing of the algorithm was evaluated to determine whether age information for subjects in the validation dataset was reported and, if so, whether the dataset included pediatric patients. Two investigators (R.B. and M.N.) independently extracted all data, with resolution of discrepancies by a third investigator (F.B.). This study adhered to Strengthening the Reporting of Observational Studies in Epidemiology (STROBE) reporting guidelines.

## Results

A total of 876 AI/ML-enabled medical devices were analyzed. Of these, 549 devices (62.7%) were authorized after 2021, and 853 (97.4%) were authorized through the 510(k) clearance pathway (Figure, Table). The most common medical specialties were radiology (667 [76.1%]), cardiovascular use (91 [10.4%]), and neurology (31 [3.5%]). Age information for patients in the validation datasets was reported for 242 devices (27.6%).

**Figure.**
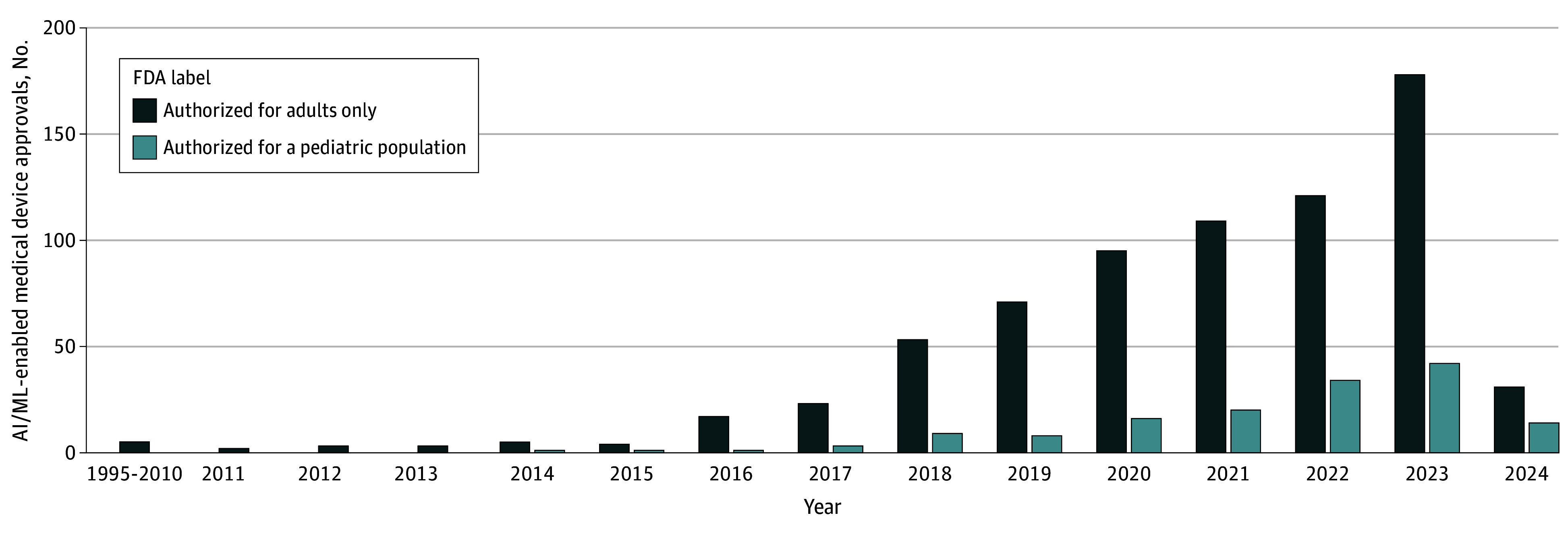
Trends in Annual US Food and Drug Administration (FDA) Authorization of Artificial Intelligence/Machine Learning (AI/ML)-Enabled Medical Devices

**Table.** Characteristics of Artificial Intelligence/Machine Learning (AI/ML)-Enabled Medical Devices Authorized by the US Food and Drug Administration (FDA)

Characteristic	Medical devices, No. (%)		
	Overall (N = 876)	Authorized for a pediatric population (n = 149)	Authorized for adults only (n = 727)
Authorization characteristics			
FDA approval pathway			
510(k) Clearance	853 (97.4)	146 (98.0)	707 (97.2)
De novo	20 (2.3)	3 (2.0)	17 (2.3)
Premarket approval	3 (0.3)	0	3 (0.4)
Medical specialty			
Radiology	667 (76.1)	119 (79.9)	548 (75.4)
Cardiovascular	91 (10.4)	10 (6.7)	81 (11.1)
Neurology	31 (3.5)	13 (8.7)	18 (2.5)
Hematology	17 (1.9)	1 (0.7)	16 (2.2)
GI/GU	13 (1.5)	0	13 (1.8)
Other^a^	57 (6.5)	6 (4.0)	51 (7.0)
Characteristics of validation dataset			
Includes information on patient ages	242 (27.6)	54 (36.2)	188 (25.9)
Includes pediatric patients			
Yes	40 (4.6)	28 (18.8)	12 (1.7)
No	187 (21.3)	22 (14.8)	165 (22.7)
Unknown^b^	649 (74.1)	99 (66.4)	550 (75.8)

Abbreviation: GI/GU, gastrointestinal/genitourinary.

^a^ Other includes the following specialties with fewer than 10 device authorizations: anesthesiology; clinical chemistry; dental; ear, nose, and throat; general and plastic surgery; general hospital; hematology; immunology; microbiology; obstetrics and gynecology; ophthalmology; orthopedics; pathology; and physical medicine.

^b^ Includes devices where either age information was available, but inclusion of pediatric patients could not be determined (eg, patient ages described using mean values with standard deviations) or age information was not available.

There were 149 AI/ML-enabled medical devices labeled for pediatric use, or 17.0% of all AI/ML-enabled device authorizations. Another 292 devices (33.3%) were authorized explicitly only for adults, and 435 (49.7%) were silent on pediatric use. Most focused on radiology (79.9%), neurology (8.7%), and cardiovascular (6.7%) specialties. For 54 pediatric devices (36.2%), approval summaries described the ages of patients in the validation datasets. Overall, 28 AI/ML-enabled devices labeled for pediatric patients (18.8%) reported using validation datasets that included pediatric patients, while 22 devices (14.8%) were validated using adult data only, and the remaining 99 (66.4%) did not report whether pediatric patients were studied.

## Discussion

Despite rapid growth in the availability of AI/ML-enabled devices in recent years, only a small number have been authorized for pediatric use. Among devices labeled for pediatric patients, few device manufacturers disclosed information in regulatory documents on whether algorithm validation was performed in pediatric cohorts and only 18.7% explicitly described validation using datasets that included children. The current regulatory framework may expose children to off-label use, differential performance of algorithms, and safety risks.^2^ Additionally, the lack of standardized reporting of pediatric device characteristics precludes informed decision-making by health care clinicians on appropriate device use. Study limitations include reliance on a database curated from publicly available FDA information, exclusion of devices potentially missing from the FDA’s website, and inability to account for device specifications that may be published by manufacturers elsewhere.

Pediatric AI/ML-enabled devices should be validated using representative datasets and should include complete and standard documentation on pediatric testing and authorization. Such changes will require cooperation across regulatory and industry stakeholders with a commitment to safe, equitable, and effective AI/ML development for children.

## Notes

Supplement 1. Data Sharing Statement
