# Supplementary material for: US FDA Approval of Pediatric Artificial Intelligence and Machine Learning–Enabled Medical Devices
Source: JAMA Pediatr. 2024 Dec 16;179(2):212–4. doi: 10.1001/jamapediatrics.2024.5437 (PMC11791695; doi:10.1001/jamapediatrics.2024.5437)
Supplement: Supplement 1. — Data Sharing Statement [file jamapediatr-e245437-s001.pdf]

Data Sharing Statement

Brewster. US FDA Approval of Pediatric Artificial Intelligence and Machine Learning–Enabled Medical Devices. *JAMA Pediatr*. Published December 16, 2024. doi:10.1001/jamapediatrics.2024.5437

Data
Data available: No
